# Post-Ejaculatory Blood Plasma Canine Prostate-Specific Esterase Concentrations May Predict Total Motility Decline After Sperm Freezing in Dogs

**DOI:** 10.3390/ani16050755

**Published:** 2026-03-01

**Authors:** Florin Petrișor Posastiuc, Guillaume Domain, Nicolae Tiberiu Constantin, Lotte Spanoghe, Joke Lannoo, Ann Van Soom

**Affiliations:** 1 Department of Internal Medicine, Reproduction and Population Medicine, Faculty of Veterinary Medicine, Ghent University, Salisburylaan 133, 9820 Merelbeke, Belgium; 2 Department of Clinical Sciences II, Faculty of Veterinary Medicine, University of Agronomic Sciences and Veterinary Medicine of Bucharest, 105 Blvd. Splaiul Independentei, 050097 Bucharest, Romania; tiberiu.constantin@fmvb.usamv.ro

**Keywords:** canine semen cryopreservation, semen freezability, canine prostate-specific esterase, sperm quality

## Abstract

Despite the widespread use of cryopreserved dog semen, reliable predictors of individual semen freezability are still not available. This study investigated the relationship between post-ejaculatory blood plasma concentrations of canine prostate-specific esterase (CPSE) and semen quality following cryopreservation. The CPSE levels were quantified using a canine-specific sandwich-type immunoassay, and sperm quality was evaluated in fresh and frozen–thawed samples using motility, kinematic, and morphological sperm parameters. Cryopreservation resulted in a significant decline in sperm motility and morphology, with marked variability in the magnitude of post-thaw changes among individuals. CPSE showed significant correlations with several morpho-functional parameters in both fresh and frozen–thawed sperm (*p* ≤ 0.05). However, its strongest predictive value for freezability was observed for total motility (*p* = 0.004), with a minimum threshold of 53 ng/mL identifying dogs showing a ≥20% post-thaw motility decline (AUC = 0.785, *p* = 0.010). These findings suggest that post-ejaculatory CPSE levels in blood plasma may reflect sperm cryoresilience and serve as a potential biomarker of canine sperm freezability.

## Simple Summary

Freezing dog semen is important for breeding and genetic preservation, but sperm from some dogs are more easily damaged by freezing than others. At present, there is no simple way to predict which dogs produce semen that freeze well. In this study, we investigated whether the amount of a protein released by the prostate gland is related to how well sperm survives freezing and thawing. We found that dogs with lower levels of this protein generally had better sperm quality, including better movement, both before freezing and after thawing. A specific blood level was identified that helped distinguish dogs whose sperm were more sensitive to freezing. These results suggest that measuring this protein in blood after ejaculation could help predict whether a dog’s semen is suitable for freezing. This information may help veterinarians and breeders improve semen freezing success and avoid unnecessary procedures.

## 1. Introduction

Over the past few years, interest in canine sperm cryopreservation has steadily increased, driven by its applications in genetic preservation, assisted reproduction, and international breeding programs [1]. Despite notable methodological advances in sperm processing and storage, post-thaw sperm quality remains highly variable among dogs, even when identical protocols are applied. This variability has highlighted the need for research into cryotolerance or freezability markers that could reliably predict how semen quality will react to the freezing process.

When standardizing the methodology behind the freezing itself, the response of spermatozoa to cryopreservation is shaped by a complex system involving intrinsic structural properties such as plasma membrane characteristics or seminal plasma composition [2]. In some canine sperm-freezing protocols, seminal plasma is removed by centrifugation, and the sperm pellet is subsequently resuspended in the chosen extender [3,4,5]. However, complete elimination of seminal plasma is not realistically achievable, as a small amount always remains in the tube after the supernatant is discarded. Moreover, even brief contact between spermatozoa and seminal plasma before centrifugation may still influence freezing tolerance, as seminal plasma exerts several actions on the cells, including antioxidant support, mitochondrial function protection, and mediation of specific interactions between spermatozoa and various proteins or membrane vesicles [2,6,7].

As previously hypothesized, the real impact of seminal plasma on sperm derives primarily from its specific constituents; therefore, its exact composition is most critical, and this composition can vary considerably between individuals [2,8]. For instance, dogs with higher concentrations of cholesterol in seminal plasma have been associated with better freezing outcomes [9]. On the other hand, much less is known about the impact of seminal plasma proteins. Proteomic data have revealed that the canine ejaculate has a complex structure, with 268 different proteins identified in both the sperm-rich and prostatic fractions, 251 of which are common to both [7]. Thus, it can be hypothesized that the prostate makes a significant contribution to the protein composition of the seminal plasma by secreting several proteins, some of which are essential for sperm function [10].

The predominant product of the canine prostate, which is also identified in the seminal plasma and prostatic cells, is an arginine esterase known as canine prostate-specific esterase (CPSE) [11,12]. Most research on CPSE has emphasized its value as a biomarker for benign prostatic hyperplasia, supporting its use in diagnosis as well as in the follow-up and assessment of therapeutic responses [13,14,15]. However, CPSE may have additional roles, as it is also a sperm-binding protein that has been identified in the post-acrosomal region and the tail of ejaculated spermatozoa [16]. The multifunctional nature of CPSE, together with its ability to interact with other seminal plasma components such as free zinc particles, suggests that it may also influence sperm quality, making it a promising factor to explore in relation to sperm cryoresilience [17]. In clinical practice, CPSE is measured during breeding soundness evaluations by determining circulating concentrations in blood serum or plasma, for which both in-house analyzers and commercial ELISA kits are available [12,14,18]. Interestingly, blood serum CPSE levels may increase shortly after ejaculation, suggesting that a transfer of CPSE from its primary location in the seminal plasma into the bloodstream may occur [19].

Therefore, the present study aimed to investigate whether post-ejaculatory blood plasma CPSE concentrations are associated with semen quality and the ability of spermatozoa to withstand freezing in clinically healthy dogs. Specifically, we sought to determine whether CPSE levels correlate with conventional semen parameters, functional sperm characteristics, and post-thaw outcomes, thereby assessing the potential value of CPSE as an indicator of freezability.

## 2. Materials and Methods

### 2.1. Ethics Statement

This study was approved by the Ethical Committee for Animal Experiments of the Faculty of Veterinary Medicine and the Faculty of Bioscience Engineering at Ghent University (protocol code 2024-087; approval date: February 2025). All procedures were conducted in accordance with institutional guidelines and relevant national and European regulations on animal experimentation.

### 2.2. Animals

Thirty clinically healthy male dogs from 21 different breeds, with a median age of 3.29 years (interquartile range [IQR] = 4.13) and a median body weight of 26.5 kg (IQR = 17.63), were enrolled in the study. All dogs underwent ultrasound examinations to rule out any gross prostatic or testicular lesions. Prostatic dimensions were obtained in longitudinal and transverse planes, allowing calculation of the actual prostatic volume using the formula proposed by Atalan et al. (1999) [20]. This value was subsequently compared with the expected prostatic volume estimated from age and body weight according to the formula of Ruel et al. (1998) [21]. In all dogs, the actual prostatic volume was lower than the expected value, thereby excluding prostatomegaly according to a previously described approach [18].

### 2.3. Semen Collection and Cryopreservation

The sperm-rich fraction was collected by digital manipulation into sterile plastic vials, placed in an incubator at 37 °C and immediately analyzed.

Following semen quality assessment, cryopreservation was done using the two-step Uppsala method [22], according to the adapted protocol described by Domain et al. (2022) [3]. Briefly, the samples were centrifuged at 720× *g* for 5 min (22 °C), in order to remove seminal plasma. The sperm pellets were diluted with a TRIS–citric acid–fructose extender containing 20% egg yolk and 3% glycerol to obtain a concentration of 200–400 × 10^6^ spermatozoa/mL. After a 2 h equilibration at 5 °C, the samples underwent a second dilution with an extender containing 7% glycerol and 1% Equex STM paste, resulting in a final concentration of 100–200 × 10^6^ spermatozoa/mL. Subsequently, the extended sperm was loaded in 0.5 mL straws and frozen with a constant-rate freezing device (IceCube 1810, SyLab, Purkersdorf, Austria) following the previously described curve by Schäfer-Somi et al. (2006) and stored in liquid nitrogen at −196 °C [23].

### 2.4. Semen Analysis

The analyses were performed twice on each sample: first on the fresh semen within 5 min following semen collection, and again after ≥24 h of cryogenic storage. The cryopreserved straws were thawed in a 37 °C water bath for 30 s and then transferred to an incubator set to the same temperature for an additional 5 min, before being analyzed.

Semen volume was measured, and sperm concentration was assessed in fresh semen samples using a Nucleocounter-SP100 system (ChemoMetec A/S, Allerød, Denmark). The obtained values were used to calculate total sperm output (TSO) by multiplying the measured sperm concentration by the volume of the second (sperm-rich) fraction.

Motility and kinematic parameters were assessed in both fresh and thawed samples using a computer-assisted sperm analysis system (ISAS^®^v1, Proiser, Valencia, Spain) with a heated stage at 37 °C and a 10× negative phase-contrast objective. For each analysis, samples were diluted to a concentration of 30–60 × 10^6^ cells/mL with a 37 °C preheated TRIS–citric acid–fructose thawing medium [22]. A 4.5 µL droplet of the diluted sample was loaded on the ISAS^®^D4C20 slide (Proiser, Valencia, Spain) and 4 fields were captured and analyzed for total motility (TM, %), progressive motility (PM, %), average path velocity (VAP, µm/s), straight line velocity (VSL, µm/s), curvilinear velocity (VCL, µm/s), amplitude of lateral head displacement (ALH, μm), beat cross frequency (BCF, Hz), wobble (WOB, %), straightness (STR, %), and linearity (LIN, %). The predefined settings followed the protocol of Domain et al. (2022) with the following adaptations: forty-five consecutive, digitized images were obtained at the same frame rate of 60 fps, particle areas to be detected have been adapted to 10–80 µm^2^ and an immotile sperm cell was defined as presenting a VCL < 20 µm/s [24].

Morphology was assessed on eosin/nigrosin-stained smears under bright-field microscopy at 1000× magnification (Olympus BX51TF, Tokyo, Japan). Two hundred sperm cells were assessed per sample for 24 morphological abnormalities, grouped into head, midpiece and tail defects, and further classified as minor or major as presented in Table 1. Operational definitions of individual abnormalities were based on established criteria as described in the WHO Laboratory Manual for the Examination and Processing of Human Semen (6th edition) and the most recent standardization of dog sperm morphology classification [25,26].

Furthermore, the Teratozoospermia Index (TZI) and the Multiple Anomaly Index (MAI) were determined as previously defined [27]. The TZI was calculated by dividing the total number of morphological defects by the number of spermatozoa exhibiting at least one abnormality, whereas the MAI quantified the occurrence of multiple structural defects within individual spermatozoa, expressed as the proportion of spermatozoa presenting two or more anomalies relative to the total sperm population.

### 2.5. Evaluation of CPSE Levels

Twenty to thirty minutes after semen collection, blood samples were obtained from the cephalic vein into lithium heparin tubes (BD Vacutainer, Plymouth, UK), centrifuged at 2500× *g* for 10 min, and the resulting plasma was stored at −20 °C until analysis. CPSE levels were determined using a canine-specific sandwich-type immunoassay kit (CPSE ELISA Kit EK762313, AFG Bioscience LLC., Northbrook, IL, USA) as previously described [18]. In brief, standards were serially diluted and 50 µL of each standard was added in duplicate to a 96-well plate. Plasma samples (10 µL) were combined with 40 µL of diluent per well. Following incubation at 37 °C for 40 min, the plate was washed and incubated with 50 µL of horseradish peroxidase-conjugated antibody (excluding blanks). After a further wash step, chromogenic substrates A and B (50 µL each) were added, and the plate was incubated in the dark for 20 min. The reaction was stopped with 50 µL of stop solution, and absorbance was read at 450 nm using a Multiskan™ GO spectrophotometer (Thermo Fisher Scientific, Vantaa, Finland). The assay detection limit was 0.31 ng/mL, with intra- and inter-assay coefficients of variation of ≤8% and ≤10%.

### 2.6. Statistical Analysis

Data were analyzed in IBM SPSS Statistics 26.0 (IBM Corp., Armonk, NY, USA). Normality was evaluated with the Shapiro–Wilk test (α = 0.05). Given the non-normal distribution of the variables, the data are reported as the median and IQR. Differences between fresh and post-thaw sperm parameters were assessed using the Wilcoxon signed-rank test. To assess the magnitude of cryopreservation-induced changes and interindividual variability, differences between fresh and post-thaw values were calculated for motility, kinematic, and morphological parameters and, where relevant, described using median, IQR, and range (minimum–maximum). Associations between CPSE levels and semen parameters from fresh and post-thaw samples, and their respective differences, were evaluated using Spearman’s rank correlation. For TM, linear regression evaluated the predictive value of CPSE, and receiver operating characteristic (ROC) analysis determined its ability to identify dogs with a ≥20% TM decline post-thaw and the optimal cutoff value. Statistical significance was set at *p* ≤ 0.05.

## 3. Results

The median CPSE level was 51.33 ng/mL (IQR = 17.11) and showed no significant correlation with the volume of the second fraction, concentration or TSO (*p* > 0.05).

Median values and IQRs for the evaluated motility and kinematic parameters in both fresh and post-thaw samples are presented in Table 2, while sperm morphology data are shown in Table 3.

Cryopreservation resulted in a significant reduction in TM, PM, VCL, VSL, VAP, LIN, STR, WOB, and BCF when compared with fresh semen samples (*p* ≤ 0.001 for all parameters, except BCF: *p* ≤ 0.01). In contrast, no significant difference was observed for ALH between fresh and post-thaw samples (*p* = 0.649).

Significant differences were observed between fresh and post-thaw semen samples for several morphology parameters. The proportion of spermatozoa with normal morphology was significantly lower in post-thaw samples (*p* ≤ 0.001), while the proportion of abnormal spermatozoa was significantly higher (*p* ≤ 0.001). Head defects increased significantly after thawing (*p* ≤ 0.001), whereas no significant difference was detected for midpiece defects (*p* = 0.657). A modest but significant increase was observed for tail defects in post-thaw samples (*p* ≤ 0.05). The rise in head defects was primarily attributable to higher frequencies of nuclear vacuoles, amorphous heads, and detached acrosomes (all *p* ≤ 0.001), while the rise in tail defects was mainly associated with an increased incidence of bent tails (*p* ≤ 0.01).

The median TZI in fresh semen samples, although slightly higher (1.54; IQR = 0.49), did not differ significantly from the value observed in post-thaw samples (1.47; IQR = 0.48; *p* = 0.574). A similar pattern was observed for MAI, which also showed no significant difference between fresh (0.15; IQR = 0.10) and post-thaw semen (0.19; IQR = 0.12).

The magnitude of cryopreservation-induced changes varied widely among individuals. The decline in TM ranged from 3.60% to 48.40%, with a median (IQR) of 22.70% (14.40%), while the reduction in PM ranged from 14.30% to 60.20%, with a median (IQR) of 29.30% (13.65%). Similarly, marked variability was observed for kinematic parameters, with reductions in VSL ranging from 14.80 to 62.32 µm/s (median [IQR]: 32.88 [19.81]) and in STR ranging from 2.12% to 26.20% (median [IQR]: 11.68 [4.59]). Changes in the proportion of morphologically normal sperm ranged from −5.00% to 18.50%, with a median (IQR) of 8.00% (6.63%), further indicating pronounced interindividual differences in the response to freezing and thawing.

In fresh semen, CPSE was negatively correlated with TM (ρ = −0.431, *p* ≤ 0.05), VCL (ρ = −0.511, *p* ≤ 0.01), VSL (ρ = −0.590, *p* ≤ 0.01), and VAP (ρ = −0.642, *p* ≤ 0.001). In post-thaw samples, similar negative associations were found with TM (ρ = −0.701, *p* ≤ 0.001), PM (ρ = −0.641, *p* ≤ 0.001), VCL (ρ = −0.438, *p* ≤ 0.05), VSL (ρ = −0.395, *p* ≤ 0.05), VAP (ρ = −0.455, *p* ≤ 0.05), and BCF (ρ = −0.400, *p* ≤ 0.05).

CPSE levels were also significantly associated with sperm morphology, showing negative correlations with the proportion of morphologically normal sperm in both fresh (ρ = −0.483, *p* ≤ 0.01) and post-thaw samples (ρ = −0.405, *p* ≤ 0.05), and positive correlations with the proportion of major defects (fresh: ρ = 0.520, *p* ≤ 0.01; post-thaw: ρ = 0.467, *p* ≤ 0.01).

When the magnitude of cryopreservation-induced changes was specifically evaluated, CPSE concentrations were only significantly associated with the reduction in total motility. A moderate positive correlation was observed between circulating CPSE levels and the decline in total motility following cryopreservation (ρ = 0.529, *p* ≤ 0.01), indicating that higher CPSE concentrations were associated with greater post-thaw motility loss. No significant associations were detected between CPSE levels and the cryogenic reduction in the other motility, kinematic, or morphological parameters.

**Figure 1:**
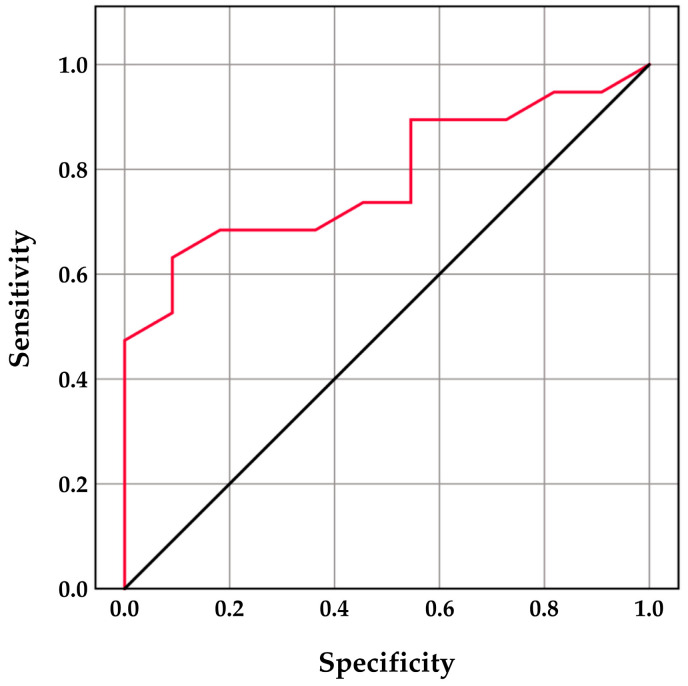
Receiver operating characteristic (ROC) curve illustrating the ability of blood plasma canine prostate-specific esterase (CPSE) concentrations to discriminate dogs exhibiting a ≥20% reduction in post-thaw total motility. The red line represents the ROC curve for CPSE, while the black diagonal line indicates the line of no discrimination (area under the curve = 0.5).

Linear regression analysis further confirmed a significant predictive relationship between CPSE concentrations and the extent of total motility decline, with CPSE accounting for 25.4% of the variability in post-thaw TM reduction (R^2^ = 0.254; *p* = 0.004). Receiver operating characteristic (ROC) curve analysis (Figure 1) demonstrated that CPSE concentrations were able to discriminate dogs that exhibited a ≥20% reduction in total motility after freezing. The optimal cutoff value was identified at 53 ng/mL, yielding an area under the curve (AUC) of 0.785 (*p* = 0.010), with a sensitivity of 63.2% and a specificity of 90.9%.

Neither TZI nor MAI, in either fresh or post-thaw samples, showed a significant correlation with CPSE (*p* > 0.05).

## 4. Discussion

Cryopreservation induces multiple alterations in spermatozoa as a consequence of rapid changes in osmolarity and temperature, which collectively contribute to an increase in plasma membrane fluidity [2]. As temperature decreases, membrane lipids undergo a phase transition from a liquid-crystalline to a more rigid crystalline state [28]. This is accompanied by lipid peroxidation, increased production of reactive oxygen species, and even loss of membrane lipids [28]. All of these processes are reflected in conventional semen analysis through compromised sperm motility and morphology parameters, which directly influence decision-making in breeding and cryopreservation programs [29].

In the present study, the freezing–thawing cycle resulted in a significant reduction in both total and progressive motility, as well as in most kinematic parameters and the proportion of morphologically normal spermatozoa; this is in agreement with previous reports describing comparable cryopreservation-associated changes assessed using both conventional and more advanced analytical approaches [30,31,32,33,34]. Notably, the magnitude of these cryopreservation-induced changes varied markedly among individuals, highlighting pronounced interindividual differences in sperm cryoresilience and underscoring the need for objective predictors of freezing outcome.

In this context, the present findings highlight the potential of post-ejaculatory blood plasma CPSE levels as an indicator of semen quality and a predictor of cryopreservation outcomes. As the predominant protein in canine seminal plasma, CPSE has been proposed to play a modulatory role in multiple aspects of sperm cell function, such as its zinc-binding capacities [10,12,35]. Zinc-binding proteins in seminal plasma regulate the availability of free zinc ions, which have been demonstrated to directly affect sperm motility [17,35,36]. According to the present findings, higher circulating CPSE concentrations were associated with lower TM, VCL, VSL, and VAP in fresh semen, and with further reductions in PM and BCF in frozen–thawed samples. In partial agreement with the present findings, a previous study investigating fresh canine semen reported a tendency toward reduced sperm motility in dogs with higher serum CPSE concentrations, although no associations with other semen parameters were identified [37]. Likewise, that study focused on dogs with CPSE values below the established diagnostic threshold for prostate volume increase of 90 ng/mL [38], which is comparable to the range reported in the present investigation. However, the temporal relationship between blood sampling and semen collection was not specified, making direct comparison with post-ejaculatory CPSE measurements difficult.

Previous work demonstrated that blood serum CPSE concentrations in healthy intact dogs increase significantly immediately after ejaculation (55.07 ± 22.08 ng/mL) [19], reaching values comparable to the median concentration observed in our samples collected 20–30 min post-ejaculation (51.33 ng/mL; IQR = 17.11). This post-ejaculatory rise in circulating CPSE has been attributed to a wash-back of the enzyme into the bloodstream, possibly resulting from a transient permeabilization of the haemato-prostatic barrier caused by increased prostatic blood flow or by the contractions of the pelvic and periprostatic tissues during ejaculation [19,39]. However, a greater transfer of CPSE into the bloodstream may lead to reduced availability of the enzyme in seminal plasma, where it normally binds zinc molecules [10,35]. This mechanism could underlie the association between higher circulating CPSE concentrations and the lower sperm motility observed in the present study, particularly considering that elevated levels of free zinc in seminal plasma have been shown to inhibit sperm motility, whereas a higher zinc-binding capacity may help preserve motility even under cold storage conditions [35,40,41].

Furthermore, CPSE is known to bind phosphorylcholine on the sperm plasma membrane after ejaculation, a property believed to play a modulatory role in fertilization, although the specific molecular interactions remain to be explained [10]. Phosphorylcholine-containing phospholipids are key components of the sperm plasma membrane, and their interaction with seminal plasma proteins is thought to contribute to the formation of a protein coat on the sperm surface, which has been hypothesized to play a protective role against cold shock [42]. In this context, it may be speculated that alterations in CPSE distribution between seminal plasma and the systemic circulation could partly underlie the association observed in the present study between elevated blood plasma CPSE concentrations and inferior sperm morphology after freezing.

The positive correlation between circulating CPSE concentrations and post-thaw TM reduction, together with the significant predictive value confirmed by linear regression, suggests that CPSE may reflect individual susceptibility of spermatozoa to cryo-induced motility loss. This interpretation is further supported by ROC analysis, which identified a post-ejaculatory blood plasma CPSE threshold of 53 ng/mL capable of discriminating dogs exhibiting a clinically relevant (≥20%) reduction in TM after freezing, with high specificity. However, given the heterogeneity of the study population with respect to age and body weight, this cut-off value should be considered preliminary and population-specific. Further validation in larger and more homogeneous cohorts will be necessary to refine its clinical applicability.

From a practical perspective, the ability to anticipate sperm cryoresilience using a readily available post-ejaculatory blood marker may offer tangible benefits in canine reproductive practice. CPSE measurement is already incorporated into breeding soundness evaluations for the assessment of prostatic function, and its additional use as a predictor of semen freezability would require no major changes to current clinical workflows. The identification of a CPSE threshold associated with a clinically relevant post-thaw decline in total motility may assist clinicians in selecting candidates for semen cryopreservation, optimizing freezing strategies, and managing expectations regarding post-thaw semen quality. Ultimately, incorporation of CPSE assessment alongside conventional semen analysis could improve decision-making efficiency, reduce unnecessary cryopreservation attempts, and enhance the overall success of canine semen freezing programs.

Several limitations of the present study should be considered. First, CPSE concentrations were measured exclusively in post-ejaculatory blood plasma, and seminal plasma CPSE levels were not assessed; therefore, any conclusions regarding CPSE redistribution between seminal plasma and the systemic circulation remain speculative. Repeated measurements before and after ejaculation would be necessary to assess individual baseline variability and post-ejaculatory dynamics. Furthermore, semen quality was evaluated using conventional methods. Morphological assessment was performed using eosin–nigrosin-stained smears under bright-field microscopy, which allows identification of gross structural abnormalities but has limited resolution for detecting subtle cryo-induced head and acrosomal alterations. Incorporation of additional functional assays, such as membrane integrity, mitochondrial activity, oxidative status, or DNA fragmentation, could provide further insight into the mechanisms linking CPSE to sperm cryotolerance. Moreover, although dogs of different breeds were included, the limited number of individuals per breed may have introduced breed-related biological variability that was not specifically addressed. Finally, future studies will be necessary to determine whether the observed associations translate into reproductive performance.

Nevertheless, the present study may represent a valuable starting point for future proteomic investigations aimed at elucidating the mechanisms through which CPSE influences sperm function and freezing tolerance.

## 5. Conclusions

The associations between post-ejaculatory circulating CPSE concentrations and post-thaw motility loss suggest that CPSE may serve as a practical biomarker of sperm cryoresilience. Although the underlying mechanisms remain to be fully elucidated, the post-ejaculatory blood plasma CPSE measurement may represent a useful addition to conventional semen analysis for predicting freezing outcomes in dogs. Further investigations into CPSE dynamics in both blood and seminal plasma are required to clarify the mechanistic basis of these observations.

## Figures and Tables

**Table 1:** Sperm morphological abnormalities assessed in canine semen samples, classified by sperm region (head, midpiece, and tail) and severity (minor or major defects).

	Head Abnormality		Midpiece Abnormality	Tail Abnormality
Minor	Macrocephaly Tapered	Fractured neck Detached acrosome	Distal protoplasmatic droplet	Bent Looped Terminally coiled
Major	Microcephaly Nuclear vacuoles Double Amorphous	Tapered Pyriform Head alone Round Knobbed acrosome	Thickened Bent Distal midpiece reflex Prox. protoplasmatic droplet	Coiled Dag defect Double

**Table 2:** Median values and interquartile ranges (IQRs) of motility and kinematic parameters in fresh and post-thaw semen samples. TM: total motility; PM: progressive motility; VCL: curvilinear velocity; VSL: straight-line velocity; VAP: average path velocity; WOB: wobble; STR: straightness; LIN: linearity; ALH: amplitude of lateral head displacement; BCF: beat-cross frequency. * Significantly different from fresh semen (Wilcoxon signed-rank test, *p* < 0.05).

	TM %	PM %	VCL µm/s	VSL µm/s	VAP µm/s	WOB %	STR %	LIN %	ALH µm	BCF Hz
	Median (IQR)									
Fresh	85.60 (9.48)	75.55 (10.23)	176.73 (32.74)	87.34 (22.52)	116.61 (21.91)	63.50 (9.68)	77.5 (4.88)	50.45 (9.88)	3.10 (0.82)	20.4 (2.97)
Post-thaw	64.40 * (23.17)	41.60 * (21.32)	142.25 * (36.12)	53.99 * (18.75)	83.84 * (22.51)	57.30 * (6.90)	65.93 * (6.74)	37.51 (8.91)	2.99 * (0.63)	18.71 * (1.91)

**Table 3:** Median values and interquartile ranges (IQRs) of sperm morphology parameters in fresh and post-thaw samples. * Significantly different from fresh semen (Wilcoxon signed-rank test, *p* < 0.05).

	Normal Morphology (%)	Head Defects (%)	Midpiece Defects (%)	Tail Defects (%)	Minor Defects (%)	Major Defects (%)
	Median (IQR)					
Fresh	74.25 (30.25)	11.25 (7.5)	11.25 (18.75)	3.25 (9.38)	10.5 (13.0)	16 (14.63)
Post-thaw	65.75 * (30.25)	21 * (9.5)	9.75 (15.38)	5.75 * (9.63)	18.5 * (11.0)	21.25 * (15.13)

## Data Availability

All data presented in this study are available upon request from the corresponding authors.

## Abbreviations

The following abbreviations are used in this manuscript:

CPSE	canine prostate-specific esterase
IQR	interquartile range
TRIS	tris(hydroxymethyl)aminomethane
TSO	total sperm output
TM	total motility
PM	progressive motility
VAP	average path velocity
VSL	straight line velocity
VCL	curvilinear velocity
ALH	amplitude of lateral head displacement
BCF	beat cross frequency
FPS	frames per second
ELISA	enzyme-linked immunosorbent assay
TZI	teratozoospermia index
MAI	multiple anomaly index
ROC	receiver operating characteristic
AUC	area under the curve
